# The Photocatalytic Degradation of Vehicle Exhausts by an Fe/N/Co–TiO_2_ Waterborne Coating under Visible Light

**DOI:** 10.3390/ma12203378

**Published:** 2019-10-16

**Authors:** Huiyun Xia, Guanyu Liu, Rui Zhang, Lifang Song, Huaxin Chen

**Affiliations:** Engineering Research Center of Transportation Materials of Ministry of Education, School of Materials Science and Engineering, Chang’an University, Xi’an 710064, China; lgy16602912220@163.com (G.L.); dykcsjzr@163.com (R.Z.); slf@chd.edu.cn (L.S.)

**Keywords:** photocatalytic degradation, doped nano-TiO_2_, vehicle exhaust, waterborne acrylic coating, visible light

## Abstract

Based on the three-dimensional network structure of a polymer and the principle of photocatalysts, a visible-light-responsive and durable photocatalytic coating for the degradation of vehicle exhaust (VE) has been constructed using a waterborne acrylic acid emulsion as the coating substrate; Fe/N/Co–TiO_2_ nanoparticles (NPs) as photocatalytic components; and water, pigments, and fillers as additives. The visible-light-responsive Fe/N/Co–TiO_2_ NPs with an average size of 100 nm were prepared by sol-gel method firstly. The co-doping of three elements extended the absorption range of the modified TiO_2_ nanoparticles to the visible light region, and showed the highest light absorption intensity, which was confirmed by the ultraviolet-visible absorption spectra (UV-Vis). X-ray diffraction (XRD) measurements showed that element doping prevents the transition from anatase to rutile and increases the transition temperature. TiO_2_ was successfully doped due to the reduction of the chemical binding energy of Ti, as revealed by X-ray photoelectron spectroscopy (XPS). The degradation rates of NO_X_, CO, and CO_2_ in VE by Fe/N/Co–TiO_2_ NPs under visible light were 71.43%, 23.79%, and 21.09%, respectively. In contrast, under the same conditions, the degradation efficiencies of coating for VE decreased slightly. Moreover, the elementary properties of the coating, including pencil hardness, adhesive strength, water resistance, salt, and alkali resistance met the code requirement. The photocatalytic coating exhibited favorable reusability and durability, as shown by the reusability and exposure test.

## 1. Introduction

With the rapid development of highway transportation and urban construction in the world, vehicle exhaust (VE) has gradually become the main source of air pollution in the world [1,2,3]. During recent decades, with the control of policies, the emission of air pollution in the world was considerably reduced [4,5]; however, the concentration of air pollutants is still high, especially in traffic-intensive urban areas [6,7,8,9]. At the same time, hazardous substances, such as CO, CO_2_, NO_x_, HC and other components in the VE cause haze and photochemical smog, both of which seriously damage human health [10,11,12,13,14]. 

At present, the methods commonly used to degrade VE mainly include physical adsorption [15], catalytic degradation [16,17,18,19], and soil VE purification [20,21]. Because of the chemical and thermal stability, high refractive index, nontoxicity, and wide band gap energy of TiO_2_ [22,23,24], it has been used as one kind of light harvester in the areas of air purification, water treatment, and deodorization [13]. Moreover, TiO_2_ was adopted as a photocatalytic material to purify VE in the areas of ceramics, concrete, and pavement [16,17]. In 1999, TiO_2_ was first used in cement pavement to degrade NO_x_ discharged from automobiles in Japan, and the results showed that TiO_2_ could purify NO_x_ in VE [19]. Leng and Yu [9] coated TiO_2_ particles on an asphalt pavement surface to purify NO_x_ in VE, and the durability of coating was evaluated. It is worth mentioning that in most of the above studies, TiO_2_ particles were incorporated into the asphalt and cement concretes to achieve efficient degradation of VE. However, this method is only applicable to new sections of road and buildings, not to existing sections. Therefore, its scope of application is limited.

As a kind of functional material, photocatalytic coatings have the advantages of simple construction, low cost, wide application, and high photocatalytic degradation of pollutants. Xu et al. [25] prepared a photocatalytic coating using an anti-corrosion coating with TiO_2_ and found that it had the effect of degrading Pirola et al. [26] prepared composite photocatalytic materials based four different matrix materials and coated them on the exterior walls of buildings. The degradation ability of silicate photocatalysts after one year is lower than that of siloxane photocatalysts. Martinez et al. [27] reported NO degradation properties with a polymer coating carrying TiO_2_ nanoparticles. However, few studies have reported the application of photocatalytic coatings in some special environments, such as tunnels with high VE concentration and weak internal light intensity [28].

Gallus et al. [29] reported three different methods to quantify the ability of photocatalytic coatings to degrade tunnel pollutants. Coating degradation efficiency was measured before or after application, upwind or downwind of the test section, and with UV lamps on or off. Guerrini [30] found that the concentration of NO_x_ in the same position decreased by more than 50% compared with that before and after coating. This study demonstrates the feasibility of photocatalytic coatings used to degrade exhaust gas in tunnels. However, an ultraviolet light source was used as the excitation source in the above studies of tunnels; in fact, only visible light is available in tunnels at present. Therefore, the degradation effect and performance of the photocatalytic coatings need to be further studied.

The main objective of this research was to develop a photocatalytic waterborne coating which can degrade VE effectively under visible light, so the performance of photocatalytic coating was characterized. In this study, the visible light responsive Fe/N/Co–TiO_2_ photocatalyst was prepared by the sol-gel method. The effect of doping elements on crystal structure and photocatalytic ability was studied. A visible-light-responsive and durable photocatalytic coating was constructed by using acrylic acid waterborne emulsion as the coating substrate; Fe/N/Co–TiO_2_ nanoparticles as photocatalytic components; and water, pigments, and fillers as additives. The photocatalytic performance of the coating under ultraviolet and visible light was characterized by self-made degradation test chamber. The reusability of VE degradation was studied by cyclic degradation test and durability test.

## 2. Experimental

### 2.1. Materials and Reagents

Titanium (IV) butoxide (TBOT, 99%) was purchased from Tianjin Kemiou Chemical Reagent Co., Ltd. (Tianjin, China). Absolute ethanol (99%) and ammonia solution (NH_4_OH, 28%) was purchased from Tianjin Fuyu Fine Chemical Co., Ltd. (Tianjin, China). Nitric acid (HNO_3_, 65%) was purchased from Chengdu Kelon Chemical Reagent Factory (Chengdu, China). Ferric nitrate (Fe(NO_3_)_3_·9H_2_O, 98%) and cobalt nitrate (Co(NO_3_)_2_·6H_2_O, 99%) were purchased from Sinopharm Chemical Reagent Co., Ltd. (Shanghai, China). Urea (99%) was purchased from Tianjin Fuchen Chemical Reagent Factory (Tianjin, China). Sodium hydroxymethyl cellulose (99%), hexametaphosphate (99%), talcum powder (99%), and barium sulfate (99%) were purchased from Tianjin Guangfu Fine Chemical Research Institute (Tianjin, China). Rutile titanium dioxide (94%) was purchased from Shanghai Coking Group Titanium Dioxide Factory (Shanghai, China). Acrylic emulsion was purchased from Guangzhou Rongdong Chemical Co., Ltd. (Guangzhou, China).

### 2.2. Preparation of Fe/N/Co–TiO_2_

Fe/N/Co–TiO_2_ catalyst samples were synthesized using the sol-gel method. Specific steps are as follows:

Firstly, 140 mL anhydrous ethanol was blended with 10 mL glacial acetic acid in a 500 mL glass beaker at room temperature. Then, 30 mL of TBOT was slowly added while stirring was continued for 20 min.

Secondly, the pH of the above mixture was adjusted to 2 by adding 3 mL nitric acid; after that, 60 mL of deionized water was added. The modified TiO_2_ sol was prepared by adding 1.527 g of Fe(NO_3_)_3_, 2.574 g of CO(NH_2_)_2_, and 2.502 g of Co(NO_3_)_2_, and stirring at a high speed for 30 min. The sol was aged for 3 days at room temperature to prepare gel.

Thirdly, the prepared sol was added to the flask and placed in a rotary evaporator to evaporate the solvent completely. The solid gel was dried in a blast drying oven at 100 °C to obtain a dry gel, and then it was ground into powder.

Finally, the prepared powder was calcined at different calcination temperatures (550 °C, 600 °C, and 650 °C) for 2 h in the muffle furnace to obtain Fe/N/Co–TiO_2_.

According to the above method, Fe–TiO_2_ was synthesized by adding an appropriate amount of Fe(NO_3_)_3_. Fe/N–TiO_2_ was synthesized by adding appropriate amount of Fe(NO_3_)_3_ and CO(NH_2_)_2_ in the preparation of modified sol. TiO_2_ was synthesized by the same preparation method without doping.

### 2.3. Preparation of the Photocatalytic Coating

Firstly, 0.5 g of sodium carboxymethylcellulose was dispersed in 25 mL water to form an aqueous solution. After adding 10 g of Fe/N/Co–TiO_2_, 10 g of rutile TiO_2_, 2.5 g of BaSO_4_, 2.5 g of talcum powder, and 0.5 g of hexametaphoshate, the paint disperser, were used for dispersion for 1 h at 30 rpm. The mixture was called liquid A. Secondly, 40 g waterborne acrylic emulsion was blended with 5 g of 2,2,4-trimethyl 1,3-pentanediol monoisobutyrate, and the pH of mixture was adjusted to 7–8 by adding aqueous ammonia solution; the above mixture was named liquid B. Thirdly, the coating was prepared by mixing liquids A and B with a small amount of defoamer and a leveling agent at 18 rpm for 0.5 h continuously. Finally, the coating was screened by a 200 mesh screen, then coated on the surface of q non-cotton fiber cement board (150 mm × 70 mm), and dried at room temperature.

### 2.4. Characterization

Colloidal particle sizes of samples were detected by Zetasizer nanoparticle size analyzer (Malvern Zetasizer Nano, Malvern, UK). The crystalline phases of samples were identified by X-ray diffraction analysis (Bruker AS, Inc., D8ADVANCE, Karlsruhe, Germany) with Cu-Kα operating at 40 kV and 40 mA. The scan rate was 0.2°/s and in the range of 15°–80°.The morphology of sample was observed using transmission electron microscopy (TEM, JEM-2100F, JEOL, Tokyo, Japan) with a test voltage of 200 kV. The TEM samples were prepared by sonication of the photocatalyst powders in ethanol for 15 min, and subsequently, dropping the dispersion onto carbon copper grids. The chemical bonding state of samples was measured by XPS (AXIS ULTRA, KRATOS, Manchester, UK) with a monochromatic Al-Kα X-ray source (KE = 1486.6 eV, 150 W). All XPS spectra were corrected by the C1s peak of external hydrocarbon contamination located at 284.8 eV. The UV-Vis diffuse reflectance spectra were measured by UV-Vis spectrophotometer equipped with an integrated sphere (UV 3600, Shimadzu Corporation, Kyoto, Japan). The reference material of the sample was BaSO_4_ and the test wavelength range was 200–800 nm.

### 2.5. Photocatalytic Degradation Experiment

The photocatalytic performances of Fe, Fe/N, and Fe/N/Co–TiO_2_ were measured by the degradation of methylene blue (MB) and VE. The photocatalytic performance of the coating was measured by VE degradation experiment.

#### 2.5.1. The Photocatalytic Degradation Experiment with MB

Firstly, 100 mg of the prepared catalyst was added to 100 mL of a 10 mg/L MB solution in a 250 mL double-layered beaker, which could be passed into cooling water to maintain the reaction temperature. The mixture was stirred in the dark for 30 min to adsorb MB. Next, photocatalytic degradation was initiated by turning on a 300 W xenon lamp that filtered out ultraviolet rays. In total, 3 mL of the reaction solution was placed in a centrifuge tube, and centrifuged for 8 min at 3000 rpm. The upper supernatant was separated and the data of MB absorbance were recorded by UV spectrometer at a wavelength (λ) of 650 nm. The degree of MB absorbance decrease was regarded as the evaluation index of photocatalytic degradation ability, which was calculated by Equation (1).

(1) $$\eta \;=\;\frac{C_{0}\;-\;C}{C_{0}}\;\times \;100\%\;=\;\frac{A_{0}\;-\;A}{A_{0}}\;\times \;100\%$$

where *η* is the degradation efficiency of MB, *C*_0_ is the initial concentration of MB, and *C* is the residual concentration after photocatalytic reaction.

#### 2.5.2. The Photocatalytic Degradation Experiment of VE

**Figure 1:**
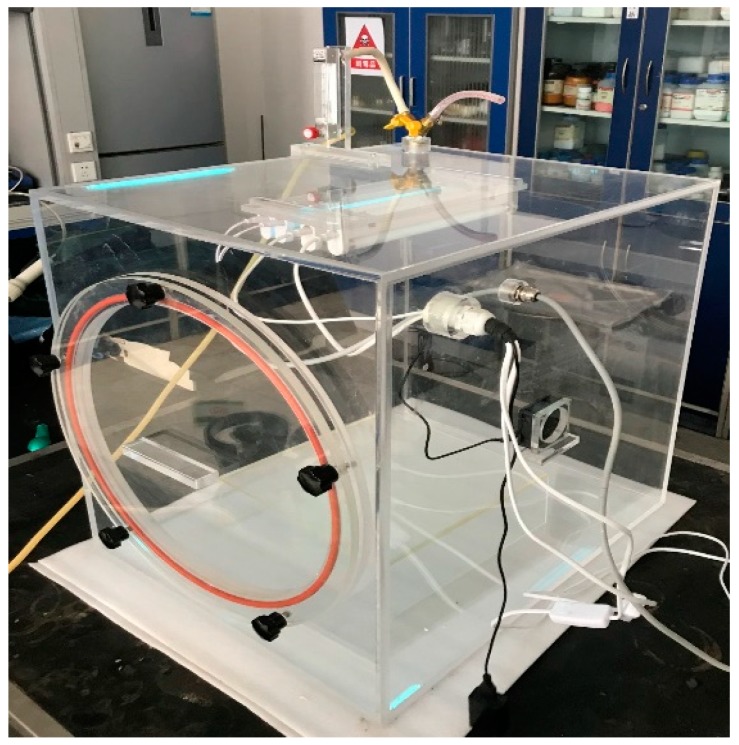
The photocatalytic vehicle exhaust (VE) reaction chamber.

The photocatalytic degradation experiment of VE was carried out by a self-made photocatalytic VE reaction chamber (Figure 1) at room temperature. The VE was from a professional preparation of Jining Xieli Special Gas Co., Ltd. (Xi’an, China) The reaction chamber was made of plexiglass, equipped with fans for dispersing gases, ultraviolet and visible light sources, and a platform for loading samples. The air tightness of the reaction chamber was ensured during the experiment, otherwise the experimental results would have been affected. The NHA-506 VE analyzer manufactured by Nantong Huapeng Electronics Co., Ltd. (Nantong, China) was used in the experiment. To ensure the accuracy of the experimental results, test errors of VE reaction chamber and VE analyzer were calibrated. 

Firstly, 2.0 g of Fe/N/Co–TiO_2_ was evenly distributed in ethanol, and the above dispersions were placed in four, 95 mm Petri dishes and dried in an oven. Next, the prepared Fe/N/Co–TiO_2_ or coating sample plate (8 pieces) was placed in the reaction chamber. The reaction chamber was sealed with a sealed cover and shielded it to avoid the Fe/N/Co–TiO_2_ or coating being irradiated by external light source. Leakage detection was performed before VE was introduced into the closed reactor. Subsequently, VE was introduced until the desired concentration was reached and the fan was turned on to disperse the gas evenly. Finally, UV or a visible light source in the reaction chamber was turned on, and the concentration of each component (including CO, CO_2_, and NO_x_) was recorded every 20 min by NHA-506 VE analyzer. The photocatalytic degradation abilities of the samples were evaluated by the degradation rates of VE’s concentration in the reaction chamber. The formula used was the same as that of Equation (1).

## 3. Results and Discussion

### 3.1. The Crystal Structures and Optical Properties of the Photocatalysts

#### 3.1.1. The Effect of Doping Elements on Colloidal Particle Size

**Figure 2:**
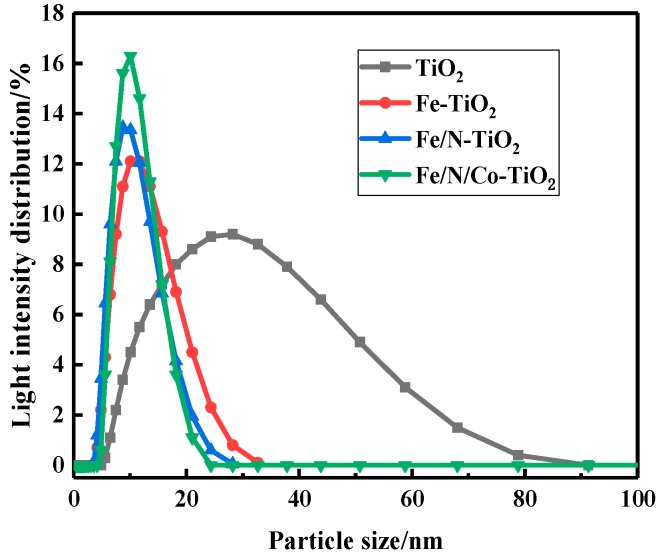
The particle size distribution of doped-TiO_2_ with different doping elements.

To investigate the effect of element species on the particle size distribution of colloidal Fe/N/Co–TiO_2_, modified TiO_2_ nanoparticles (NPs) doped with different elements were prepared, and their particle size distributions are shown in Figure 2. Compared with the TiO_2_ colloid, the particle size distributions of Fe–TiO_2_, Fe/N–TiO_2_, and Fe/N/Co–TiO_2_ colloids became narrow; the average particle sizes of sols were 11.15, 9.56, and 10.64 nm, respectively, as shown in Table 1. 

These phenomena may be related to Brownian motion [31]. When a certain amount of Fe^3+^ is incorporated into the TiO_2_ colloid, the replacement of Ti^4+^ by Fe^3+^ makes the Fe–TiO_2_ colloid negatively charged. The Fe–TiO_2_ colloids with the same charge have a repulsion effect, so the colloids are unlikely to coagulate and the particle size becomes smaller [32]. N was adopted as a non-metallic element to provide more negative charge, thus making the particle size smaller [33]. Since the doping of Co^2+^ breaks the charge balance again, the colloidal motion is intensified, and the colloidal particle size is increased.

#### 3.1.2. The Effect of Doping Elements on UV-Vis

**Figure 3:**
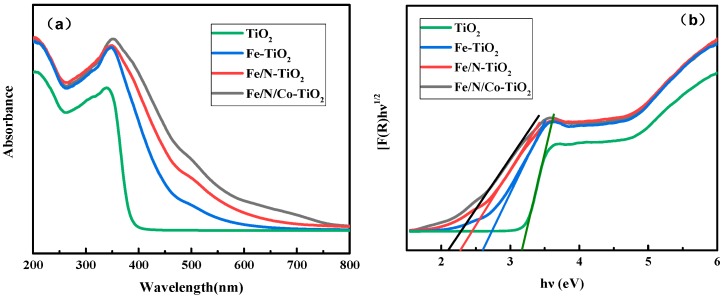
The UV-Vis absorption (**a**) and corresponding plot of transformed Kubelka–Munk function pattern (**b**) of TiO_2_, Fe, Fe/N, and Fe/N/Co–TiO_2_ particles after calcination at 650 °C.

To investigate the effect of elemental species on the optical absorption ability of Fe/N/Co–TiO_2_ NPs, the optical absorption properties of doped TiO_2_ were characterized by UV-Vis spectra. In reference to Figure 3a, it can be seen that: (i) The doping made the absorption range extend to the visible light region. (ii) The absorption intensity of doped TiO_2_ in the ultraviolet light and visible light regions increased obviously compared with un-doped TiO_2_. (iii) The Fe/N/Co–TiO_2_ showed the most obvious red shift of absorption edge and highest absorption intensity among the doped TiO_2_ NPs in the visible region. Furthermore, the band gap values of TiO_2_, Fe–TiO_2_, Fe/N–TiO_2_, and Fe/N/Co–TiO_2_ NPs were 3.2, 2.6, 2.3, and 2.1 eV estimated by Kubelka–Munk function (c.f. Figure 3b). This indicates that the band gap width decreases significantly after doping, which is more conducive to improving the visible light response’s efficiency. It can be concluded that tri-doping plays an important synergistic role in the absorption of TiO_2_ in the visible region [34].

The expansion of Fe–TiO_2_ adsorption under visible light comes from the electronic transition from the dopant energy level (Fe^3+^/Fe^4+^) to the conduction band of TiO_2_ [35,36,37]. However, the mechanisms of photoexcitation changes induced by doping with Fe or N are different. The TiO_2_ band gap narrowing is induced by the localized N 2p states (acceptor states) positioned above the TiO_2_ valence band when N is incorporated in TiO_2_ lattice [38]. The charge separation is promoted, so the band gap is further reduced. Therefore, Fe/N–TiO_2_ can be activated with much longer wavelengths than Fe–TiO_2_ [39]. It is reported that the substitution of Co^2+^ to Ti^4+^ in TiO_2_ crystals causes lattice defects and breaks the electron and hole motion states [40], which may arise from charge transfer and d–d transitions [41], leading to a further red shift. Thereby, the photoactivity of Fe/N/Co–TiO_2_ is further improved by tri-doping and exhibits more excellence in the visible region. 

#### 3.1.3. The Effect of Calcination Temperature on XRD of Fe/N/Co–TiO_2_

**Figure 4:**
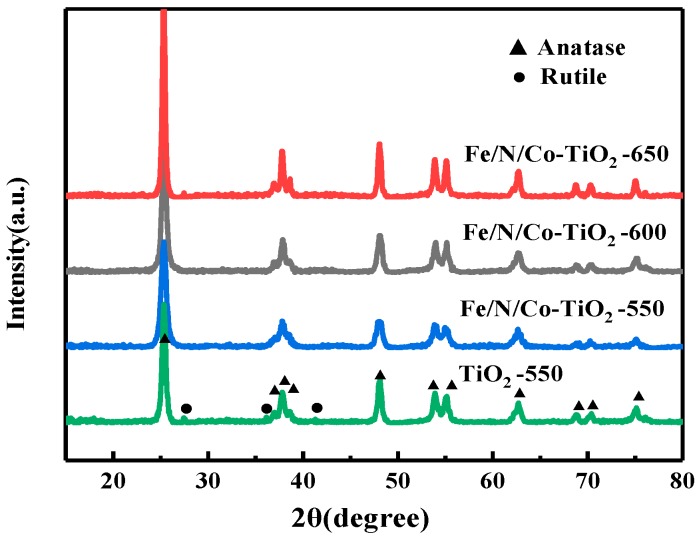
The XRD patterns of doped-TiO_2_ after calcination at different temperatures.

To investigate the effect of calcination temperature on the crystal structure, the XRD pattern of Fe/N/Co–TiO_2_ at different calcination temperatures were measured. Figure 4 shows the XRD patterns of Fe/N/Co–TiO_2_ at different calcination temperatures. The typical anatase TiO_2_ patterns appear at 2*θ* of 25.3°, 37.8°, 48.0°, 53.9°, and 62.68°, and rutile TiO_2_ appears at 2*θ* of 27.4° and 36.1°. After calcination at 550 °C, the crystal form of undoped TiO_2_ is a mixture of anatase and rutile. However, Fe/N/Co–TiO_2_ is anatase, there is no rutile. This is because the doping of Fe and N inhibits the growth of crystallite, and the transition from anatase to rutile is prevented, transition temperature increased [42,43].

The diffraction peak of anatase of Fe/N/Co–TiO_2_ became sharper as the calcination temperature increased, and the rutile peaks appeared when the calcination temperature reached 650 °C. It is also indicated that with the increase of calcination temperature, the grain growth and crystallinity increase [44]. When the calcination temperature was further increased to 650 °C, a small portion of anatase in Fe/N/Co–TiO_2_ began to be converted into a more stable rutile, which implied that the rutile transition temperature of Fe/N/Co–TiO_2_ is between 600 and 650 °C.

#### 3.1.4. The Effect of Calcination Temperature on the UV-Vis of Fe/N/Co–TiO_2_

**Figure 5:**
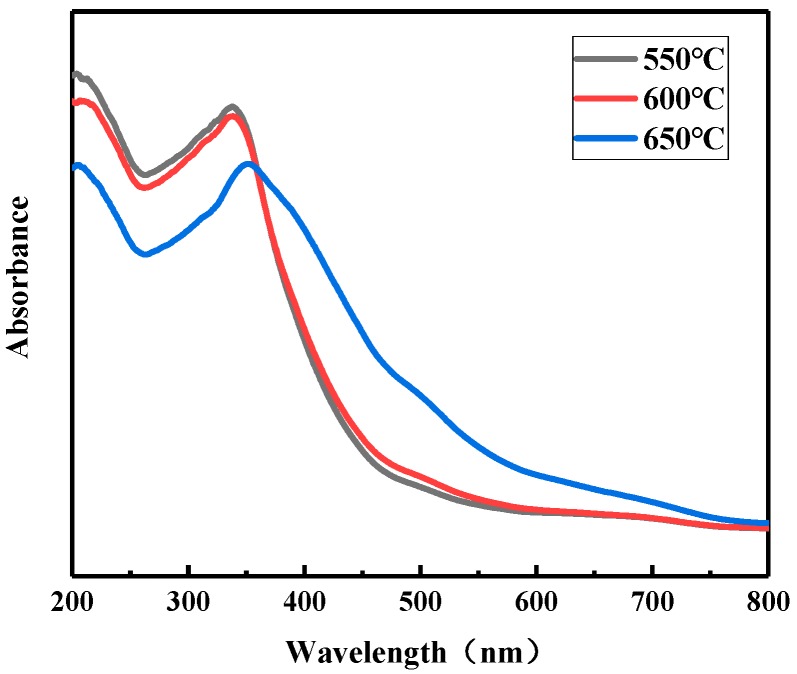
The UV-Vis spectra of Fe/N/Co–TiO_2_ after calcination at different temperatures.

Figure 5 shows the UV-Vis of Fe/N/Co–TiO_2_ prepared at different calcination temperatures. It was found that the UV-Vis spectra of Fe/N/Co–TiO_2_ prepared at 550 and 600 °C were almost the same. This is because the crystal morphology of Fe/N/Co–TiO_2_ anatase was improved, and the grain size increased with the increase of calcination temperature from 550 to 600 °C. Actually, perfect crystal form contributes to the increase of light absorption intensity, while coarse grain size inhibits the absorption and utilization of light by photocatalysts. Therefore, the absorbances of Fe/N/Co–TiO_2_ calcined at 550 and 600 °C are basically the same under the above two effects.

When the calcination temperature reaches 650 °C, the absorptive strength of Fe/N/Co–TiO_2_ in the visible region is considerably improved, while the absorptive strength in the ultraviolet region is a fair amount lower than that after calcined at 550 and 600 °C. This phenomenon may be caused by a transformation between crystal forms. Some anatase is transformed into rutile, and rutile can be excited by light of a larger wavelength range than anatase to produce photoelectrons, but anatase has both higher light absorption and photocatalytic activity than rutile [45].

#### 3.1.5. TEM of Fe/N/Co–TiO_2_

**Figure 6:**
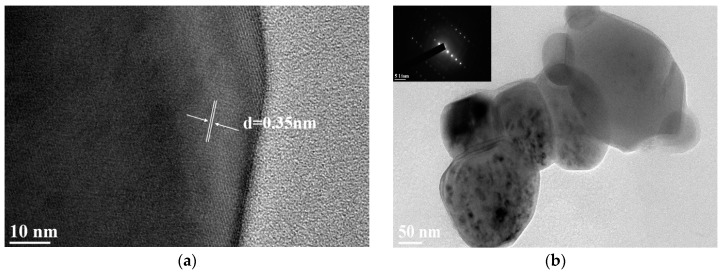
The HR-TEM (**a**) and TEM (**b**) images of Fe/N/Co–TiO_2_ particles.

Figure 6 shows the TEM images of the Fe/N/Co–TiO_2_. It can be seen that the particle size of Fe/N/Co–TiO_2_ ranges from 50 to 200 nm, and there is a certain degree of agglomeration (Figure 6a). According to the HR-TEM of Fe/N/Co–TiO_2_ displayed in Figure 6a, the d-spacing was 0.351 nm, which corresponds to the (101) lattice planes of anatase TiO_2_ [46]. Moreover, the SAED (inset) images in Figure 6b indicate the single-crystalline characteristics, which confirm that the results are consistent with XRD measurements.

#### 3.1.6. XPS Spectra of Fe/N/Co–TiO_2_

**Figure 7:**
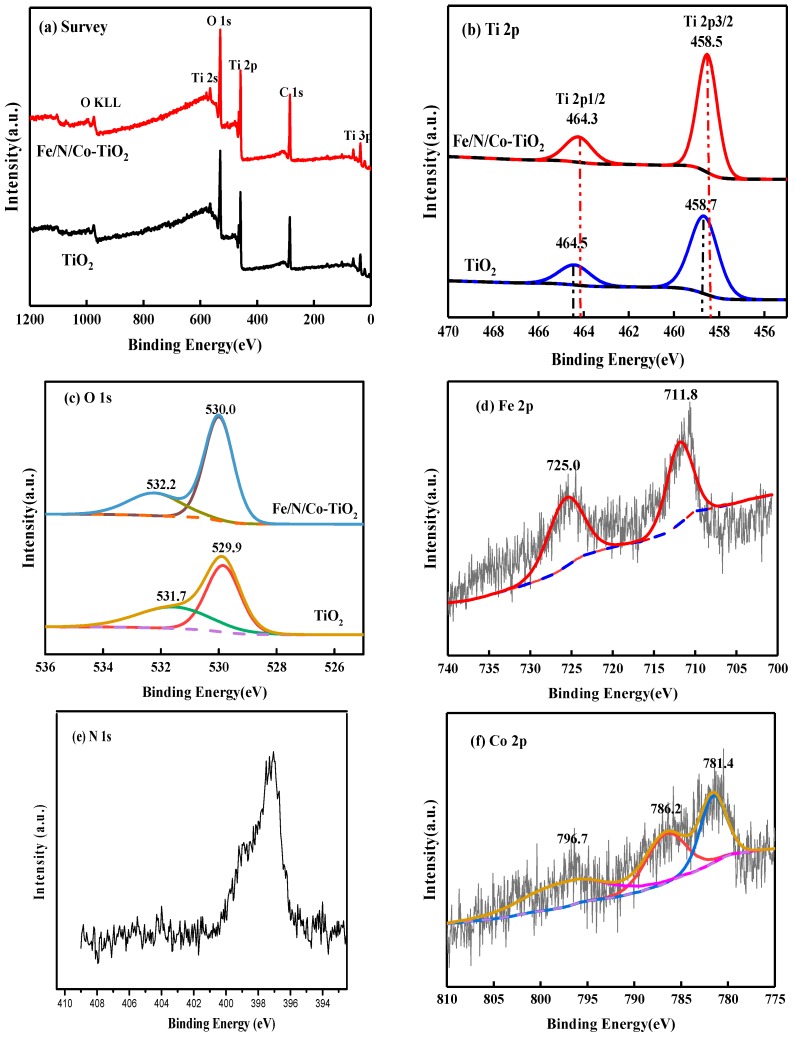
The XPS spectra: (**a**) survey, (**b**) Ti 2p, (**c**), O 1s, (**d**) Fe 2p, (**e**) N 1s, and (**f**) Co 2p of Fe/N/Co–TiO_2_ and TiO_2_ nanoparticles (NPs).

The chemical compositions of Fe/N/Co–TiO_2_ were further studied by XPS of Fe/N/Co–TiO_2_ and TiO_2_ NPs, as shown in Figure 7. The XPS of Fe/N/Co–TiO_2_ mainly contained Ti, O, C, and other elements (Figure 7a). Among them, C 1s detected by 284.8 eV was caused by organic pollutants remaining during the preparation process [47]. Ti shows a strong peak position at 458.7 and 464.5 eV, as shown in Figure 7b. The two characteristic peaks correspond to Ti2p_3/2_ and Ti2p_1/2_, respectively. The position where the peak of Ti in Fe/N/Co–TiO_2_ appears with smaller shift and is deviated by 0.2 eV, showing higher photocatalytic activity, which is consistent with the results of UV test. The characteristic peaks of O 1s are asymmetrical according to Figure 7c. There are 530.0 and 532.2 eV peaks in Fe/N/Co–TiO_2_, and 529.9 and 531.7 eV peaks in TiO_2_. The peak of O 1s at 529.9 and 530.0 eV could be attributed to the O^2−^ anions of the TiO_2_ crystalline lattice. 532.2 and 531.7 eV is the hydroxyl oxygen peak of –OH on the surface of Fe/N/Co–TiO_2_ and TiO_2_ [48]. The presence of surface –OH indicates that the Fe/N/Co–TiO_2_ has catalytic degradation ability. This is because TiO_2_ is an n-type semiconductor. When it is illuminated, the valence band electrons gain energy and jump to the conduction band to form photogenerated electrons; at the same time, holes are formed in the valence band. The –OH on the surface of TiO_2_ are easily oxidized into –OH by holes, and the –OHs have extremely strong oxidation capacity, which can oxidize inorganic substances or most organic substances into inorganic small molecules, CO_2_ and H_2_O [48]. Compared with undoped TiO_2_, the –OH peak of Fe/N/Co–TiO_2_ was stronger, and it may be inferred that it has more excellent photocatalytic degradation.

Figure 7d shows the Fe 2p spectra of Fe/N/Co–TiO_2_. The two peaks of 711.8 and 725.0 eV appeared after the peak-fit processing of Fe 2p spectra, which correspond to Fe2p_3/2_ and Fe2p_1/2_, indicating that Fe exists in the +3 valence state. In combination with the above XRD, there was no peak of Fe_2_O_3_, and the ionic radius (0.64 Å) of Fe^3+^ was similar to that of Ti^4+^ (0.68 Å) [49], so it was concluded that Fe^3+^ was successfully incorporated into the crystal lattice and formed Fe–O–Ti bonds. The XPS pattern fitting of N 1s found that only one characteristic peak appeared 397.0 eV (Figure 7e), indicating that O in a large number of N substituted TiO_2_ lattices forms Ti–N bonds [50]. There are 781.4, 786.2, and 796.7 eV peak positions after the peak-fit processing of Co 2p spectra as can be seen in Figure 7f. Among them, the peak at 781.4 and 796.7 eV correspond to Co2p_3/2_ and Co2p_1/2_ of Co^3+^ respectively. The peak at 786.2 eV corresponds to Co^2+^, and the peak of CoO at 780.0 eV did not appear [51,52], indicating that Co^3+^ and Co^2+^ coexist in Fe/N/Co–TiO_2_ and parts of Co replace Ti in the doped TiO_2_ lattice.

### 3.2. The Photocatalytic Degradation Ability of the Photocatalyst

#### 3.2.1. The Photocatalytic Degradation Performance of Fe/N/Co–TiO_2_ on MB

**Figure 8:**
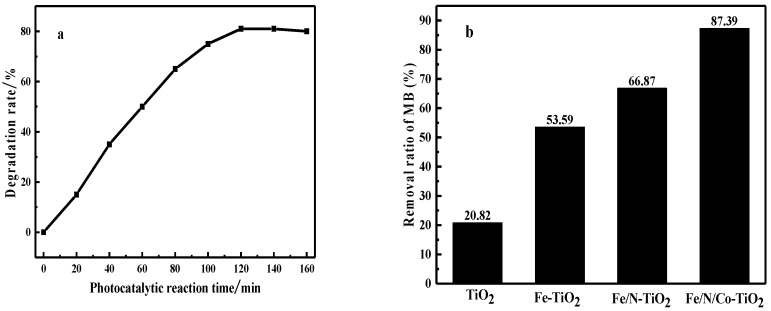
Methylene blue (MB) degradation experiment. (**a**) The variation of MB degradation efficiency with reaction time; (**b**) degradation rates for MB after 120 min under visible light irradiation.

In order to determine the photocatalytic activity of doped-TiO_2_ prepared above, the MB solution was adopted and the results are shown in Figure 8. After 120 min of irradiation, Fe/N/Co–TiO_2_ exhibited the highest catalytic degradation rate, which was 85%, followed by Fe/N–TiO2 and Fe–TiO_2_, and finally, TiO_2_. The main reasons for the improvement of photocatalytic performance may be as follows: (i) Metal/nonmetal doping improves the visible light absorption intensity of TiO_2_ [42]. (ii) The specific surface area of TiO_2_ grain may increase, the MB molecules are more easily adsorbed on the surface, and the contact area is increased, thereby the degradation rate increases [43,53].

#### 3.2.2. The Photocatalytic Degradation Performance of Fe/N/Co–TiO_2_ on VE

**Figure 9:**
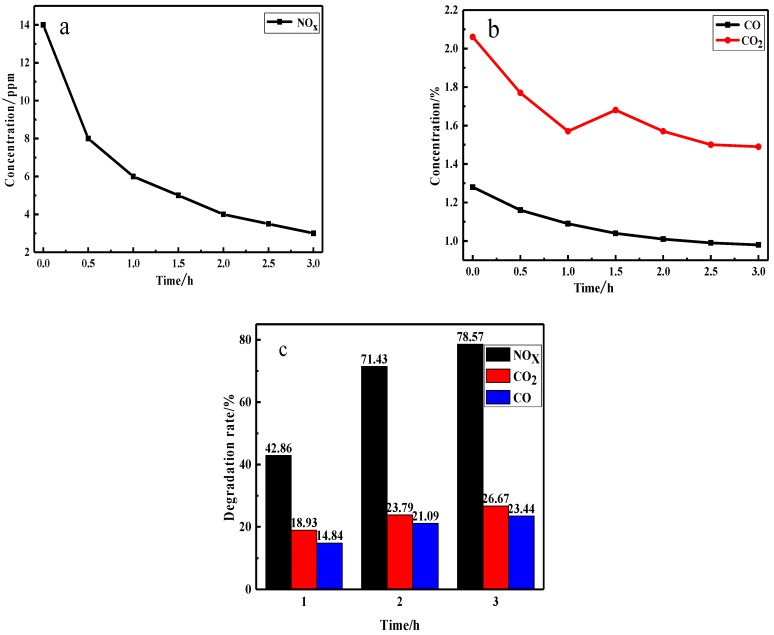
The concentrations of NO_x_, (**a**) and CO and CO_2_ (**b**) during the photocatalytic reaction process, and the photocatalytic degradation rate of VE (**c**) by doped TiO_2_ under visible light.

Figure 9 shows the photocatalytic degradation of three components in VE by Fe/N/Co–TiO_2_ under visible light. As can be seen from Figure 9a, the concentration of NOx decreased rapidly in the first half hour. After that, the concentration decline slowed down gradually. After 2 h, the concentration of NO_x_ was about 4 ppm, and the degradation efficiency was 71.04%. NO_x_ concentration decreased very slowly in the last 1 h. At the same time, the degradation efficiencies of CO and CO_2_ remained almost unchanged, as depicted in the Figure 9b. Therefore, the late degradation of VE was carried out according to the irradiation time of 2 h. The decline of degradation performance may be due to the nitric acid and nitrates produced by oxidation of NO_x_ covered with a doped-TiO_2_ surface [54]. The effective contact area between the exhaust gas and the photocatalyst was reduced. The increase in the CO_2_ concentration of the inflection point in Figure 9b may be caused by the oxidation of part of CO to CO_2_ [55].

### 3.3. The Elementary and Photocatalytic Performance of Photocatalytic Coating

#### 3.3.1. The Elementary Performance of Photocatalytic Coating

In order to ensure the effective use of photocatalytic coatings, the elementary properties of the coatings were determined according to JG/T 512-2017 (General Technical Requirements for Building Exterior Wall Coatings) first. Test results are shown in the Table 2. It was found that the elementary properties of the coating met the code requirement (JG/T 512-2017), and the resistances to water, salt, and alkali were good.

The durability of the coating can be evaluated by accelerated weather aging test (1000 h). To ensure test accuracy, three coated cotton-free fiber cement boards (labeled A, B, and C) were tested simultaneously; the results are shown in Table 3. After 6 weeks of exposure, the aging degree of the three samples can be neglected. The evaluation grade was Level 1, indicating that the photocatalytic coating meets code and construction requirements. The slight pulverization of the surface is mainly due to the photocatalytic degradation effect of the photocatalyst, which degrades the organic matter of the coating. 

#### 3.3.2. The Photocatalytic Degradation Performance of the Coating on VE

**Figure 10:**
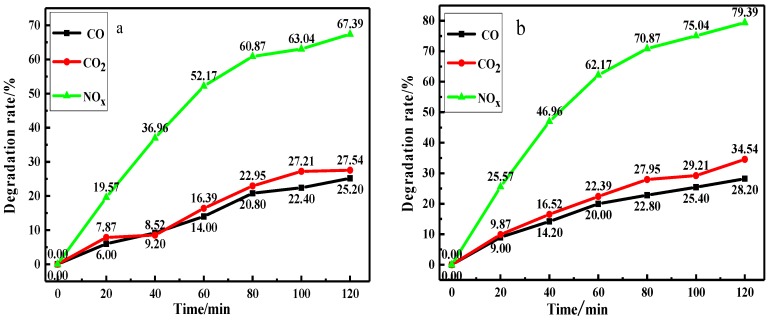
The degradation rate of VE under (**a**) visible light and (**b**) ultraviolet light.

The photocatalytic degradation performance of the coating under different illumination conditions was studied, as shown in Figure 10. The degradation rates of NO_X_, CO, and CO_2_ under visible light were 67.39%, 25.20%, and 27.54%, respectively. In contrast, under ultraviolet irradiation, the degradation efficiencies were higher, 79.39%, 28.20%, and 34.54%, respectively. This phenomenon may be due to that the absorption intensity exhibited Fe/N/Co–TiO_2_ in the ultraviolet region was higher than that of visible light (according to the UV-Vis results above). Furthermore, the energy of ultraviolet light is higher than that of visible light, and it is easier to generate more photoelectrons for photocatalytic reaction when Fe/N/Co–TiO_2_ is irradiated [56]. It is obvious that photocatalytic coating is effective at degrading the main harmful gas in VE under visible light irradiation. At the same time, the higher degradation efficiency of the photocatalytic coating is related to the network structure of the acrylic substrate, which can effectively avoid the agglomeration of doped TiO_2_ NPs [57,58].

#### 3.3.3. Reusability of the Photocatalytic Degradation

**Figure 11:**
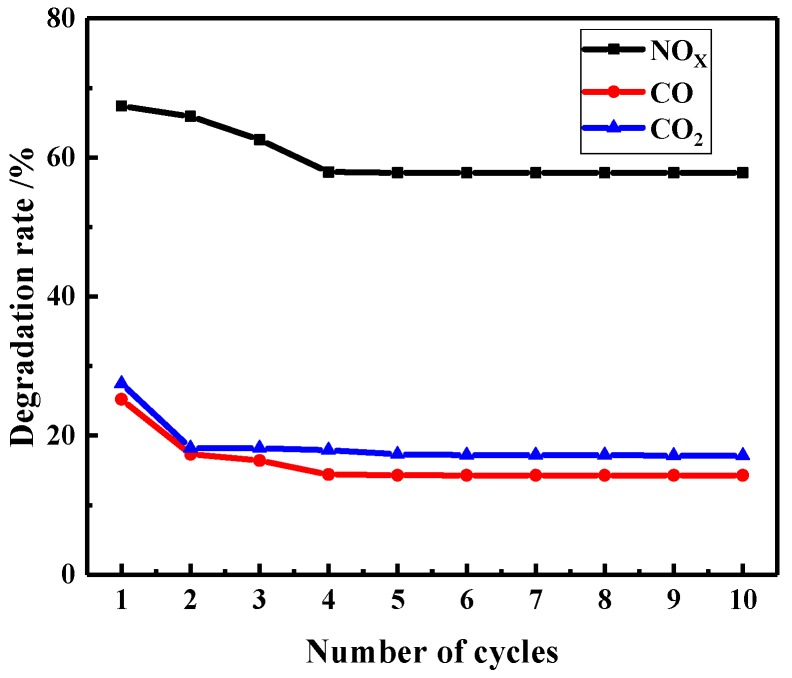
The reusability for VE degradation performance of photocatalytic coating.

Reusability is an important indicator to evaluate the performance of photocatalytic coatings. Considering that photocatalytic coatings need to be cleaned frequently in practical use, some photocatalysts may lose their activity after repeated cleaning, resulting in the reduction of photocatalytic efficiency, so the influence of rinsing times on photocatalytic properties of coatings was investigated. The specific experimental process was as follows: firstly, a VE degradation test was carried out; after that, the coating was rinsed with tap water for 1 min to ensure that the surface of the coating was clean, then dry, at room temperature. The experiments were repeated ten times and the VE degradation test results were recorded (Figure 11). 

It was found that with an increase in washing times, the degradation efficiency decreased first and then tended to be stable after four cycles. The degradation efficiencies of NO_x_, CO, and CO_2_ were slightly reduced between the first and fourth cycles. The decrease of degradation efficiency may be mainly attributed to two aspects. Firstly, with the prolongation of exposure time, as a small part of the effective photocatalytic material on the surface falls off. Secondly, during the rinsing process, a small amount of hydrophilic or water-soluble substances are not completely washed out, and remain in the pits and channels on the surface of the coating, which affects the adsorption of the coating to VE. However, after four cycles, the degradation rates of NO_x_, CO, and CO_2_ remained stable, demonstrating good reusability of the coating. This may be due to the confinement effect of the acrylic emulsion on doped TiO_2_, which does not change the dispersion state of the doped TiO_2_ on the surface of the coating during the scouring process [59]. 

#### 3.3.4. Durability of the Photocatalytic Coating

**Figure 12:**
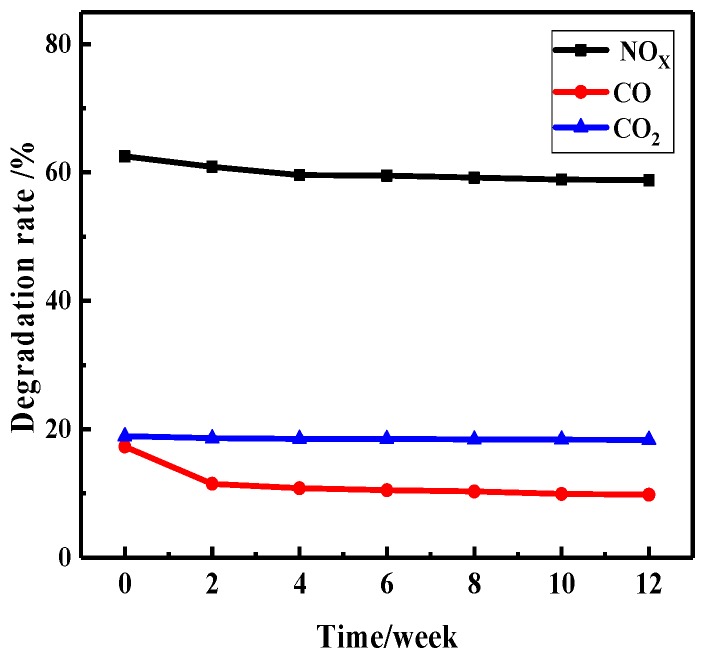
The durability for VE degradation performance of photocatalytic coating.

Durability is an important index for long-term use of coatings. It can be evaluated by the exposure test. Firstly, the photocatalytic coating was coated on the surface of the cement boards, and then the cement boards were exposed to sunlight. It was stipulated that the test site is roof, and the test day had to be sunny. Finally, VE degradation performance was measured every two weeks; test results are shown in Figure 12.

As shown in Figure 12, it can be seen that the photocatalytic coating showed a slight decrease in photocatalytic activity after natural exposure. The degradation rates of the coating on NO_x_, CO and, CO_2_ decreased by 3.7%, 7.5%, and 0.55% respectively, after 12 weeks. The main reason for this phenomenon may be that during the exposure test, the Fe/N/Co–TiO_2_ has a slight degradation effect on waterborne, acrylic substrate, resulting in a slight smashing of the surface of the coating and a small amount of photocatalyst falling off. In addition, the adsorbate on the surface of the coating may also cover the surface of the photocatalyst to affect its contact with VE [60]. However, due to the crosslinked structure of the coating and confinement effect on doped TiO_2_, most photocatalysts still adhere to the surface/interior of the coating, showing excellent durability and can meet the requirements of normal use.

## 4. Conclusions

In this study, visible light responsive Fe/N/Co–TiO_2_ nanoparticles with good photocatalytic activity were prepared. The effects of Fe, N, and Co doping on the crystal size, micro-structure, and photocatalytic performance of Fe/N/Co–TiO_2_ were investigated. The degradation rates of NO_X_, CO, and CO_2_ in VE under visible light reached 71.43%, 23.79%, and 21.09%, respectively. Then, a visible-light-responsive and durable photocatalytic coating was constructed for degrading harmful gas in the VE by loading Fe/N/Co–TiO_2_ particles into the waterborne acrylic coating. The coating exhibited efficient performance of visible light degradation for VE, and the degradation rates of NO_X_, CO, and CO_2_ reached 67.39%, 25.20%, and 27.54%. Furthermore, the photocatalytic coating exhibited favorable reusability and durability, due to the crosslinked structure in the coating and the confinement effect on doped TiO_2_. It is believed that this study provides an efficient and simple method for the purification of VE in some special environments (such as tunnels) that only have visible light.

## Figures and Tables

**Table 1:** The colloidal particle size of Fe, Fe/N, and Fe/N/Co–TiO_2_.

Number	Doping Element	Average Particle Size/nm	Distribution Coefficient (PDI)
1	Undoped	36.10	0.349
2	Fe	11.15	0.240
3	Fe/N	9.56	0.179
4	Fe/N/Co	10.64	0.204

**Table 2:** Elementary properties and corrosion resistance of photocatalytic coating.

Project	Pencil Hardness	Adhesive Strength	Water Resistance (168 h)	Salt and Alkali Resistance (168 h)
Coating	4H	1.15 Gpa (Ⅳ)	No abnormality (Ⅱ)	No abnormality(Ⅲ)

**Table 3:** The results of accelerated weather aging test (1000 h) for photocatalytic coating.

Sample	Discolouration	Pulverization	Cracking	Blistering	Damage	Evaluation Grade
A	1	1	0	0	1/S1	1
B	0	1	0	0	1/S1	1
C	1	1	0	0	1/S1	1
